# Development and Validation of Human-Computer Collaborative Classroom Second Language Learning Engagement Scale

**DOI:** 10.3390/bs16010046

**Published:** 2025-12-25

**Authors:** Yanshuang Jiang, Yuxuan Liu

**Affiliations:** 1 China Institute of Education and Social Development, Beijing Normal University, Beijing 100875, China; 11112023092@bnu.edu.cn; 2 Faculty of Education, Beijing Normal University, Beijing 100875, China

**Keywords:** second language learning, human–computer collaborative classroom, student engagement, ethnic minority students in junior high schools, scale development

## Abstract

This study developed and validated the Human–Computer Collaborative Classroom Second Language Learning Engagement Scale among 710 junior high school students studying in Mongolian. Initially, the scale’s conceptual framework was developed through a review of pertinent literature and interview, drawing on self-determination theory and socio-constructivist perspectives to define engagement in human–computer collaborative second language learning contexts. The study adopted a sequential mixed-methods design: in Phase 1, item analysis and exploratory factor analysis (EFA) were conducted using data from 437 students, resulting in a preliminary five-factor structure; in Phase 2, confirmatory factor analysis (CFA) was performed using data from the remaining 273 students to validate the factor structure. The final scale comprises five core dimensions: (1) higher-order thinking, (2) student–teacher interaction, (3) human–computer interaction, (4) active collaborative learning, and (5) learning enthusiasm. Structural equation modeling confirmed a robust five-factor model, with all fit indices indicating satisfactory model fit (e.g., CFI = 0.981, TLI = 0.977, RMSEA = 0.041). The scale demonstrates strong internal consistency (Cronbach’s α = 0.959) and construct validity. These findings highlight the reliability and efficacy of this psychometric tool for evaluating students’ engagement in second language learning within human–computer collaborative classroom environments, offering valuable insights for educators and researchers.

## 1. Introduction

The integration of intelligent technologies with collaborative pedagogies has emerged as a promising paradigm for enhancing second language (L2) acquisition, yet its application remains underexplored in specific linguistic contexts such as Mongolian-speaking students learning Chinese. Human–computer collaborative teaching—characterized by synergistic interactions between AI-driven systems and peer-based learning—has shown potential to create dynamic environments that foster student engagement (Fung et al., 2024; Andreou & Argatzopoulou, 2025). However, the absence of validated, context-responsive measurement instruments severely limits both empirical advancement and instructional optimization for this unique learner population. This study addresses this critical gap by developing and validating a comprehensive scale to assess L2 learning engagement within human–computer collaborative classrooms, thereby providing researchers and practitioners with a theoretically grounded diagnostic tool.

Grounded in self-determination theory (SDT), this research conceptualizes engagement as a multidimensional construct shaped by the fulfillment of three basic psychological needs: autonomy, competence, and relatedness (Ryan & Deci, 2020). In human–computer collaborative settings, technology uniquely scaffolds these needs by enabling personalized learning pathways (autonomy), adaptive feedback mechanisms (competence), and synchronous peer interaction platforms (relatedness) (Fung et al., 2024). Crucially, collaborative learning engagement diverges fundamentally from individual engagement; it necessitates a shift toward group cognition—the shared construction of knowledge, intellectual exchange, and co-development of problem-solving strategies among peers (Z. Guo et al., 2023). While prior investigations have examined educators’ perspectives on collaborative learning in L2 contexts (González-Lloret, 2020; Zhang et al., 2021), these studies predominantly focus on English as a Foreign Language settings without addressing technology-mediated collaboration. Existing measurement scales either assess general L2 engagement or individual computer-assisted learning, failing to capture the synergistic dynamics unique to human–computer collaborative environments.

This theoretical and empirical void is particularly acute for Mongolian-speaking Chinese learners. Current research on this population’s engagement in technology-enhanced collaborative settings remains scarce, impeding evidence-based pedagogical development. Existing instruments overlook critical cultural and linguistic dimensions—such as the pedagogical implications of linguistic distance between Mongolian and Chinese, and culturally nuanced manifestations of engagement—thereby compromising ecological validity. This paucity of validated tools not only constrains the design of responsive ICT interventions but also prevents systematic evaluation of instructional effectiveness, leaving educators without adequate diagnostic capabilities.

This study redresses these limitations through two objectives: (1) identifying core engagement dimensions in human–computer collaborative L2 classrooms via mixed-methods investigation, and (2) developing and validating a culturally responsive, psychometrically sound measurement instrument. Theoretical innovation lies in extending SDT to human–computer collaborative contexts while empirically distinguishing individual, peer-mediated, and technology-facilitated engagement components. Practical contribution includes providing a cross-sectional evaluation tool that enables researchers to assess intervention efficacy and offers educators actionable insights for optimizing task design and support structures tailored to Mongolian-speaking Chinese learners.

## 2. Literature Review

### 2.1. Human–Computer Collaborative Classroom and Second Language Learning

The advancement of AI has led to a deep integration of human–computer interaction concepts with language learning, driving innovation in language education. AI language learning tools enable personalized learning experiences, allowing teachers to tailor instruction to individual students (Rahman et al., 2024). For instance, AI-powered chatbots facilitate real-time interaction with students, providing immediate and accurate feedback on queries during language learning sessions (Y. Li et al., 2024). Additionally, chatbots assist students in navigating diverse language materials, enhancing comprehension of key concepts (Yetişensoy & Karaduman, 2024), thereby enhancing learning efficiency. The incorporation of game-based technology in the second language classroom offers a novel approach to language acquisition. By presenting engaging and challenging gaming scenarios, this method enhances students’ interest and intrinsic motivation in learning, enabling them to engage in language practice and communication within a simulated authentic linguistic environment, fostering active participation in language acquisition (Pugh-Opher, 2021). For instance, adaptive learning systems can dynamically tailor learning materials and difficulty levels to suit individual students’ needs and learning preferences, thereby personalizing a unique learning trajectory for each student. This approach not only accommodates varying learning styles among students but also cultivates a heightened sense of accomplishment and self-assurance during the learning process, consequently enhancing their educational engagement (Y. Li et al., 2024).

A human–computer collaborative classroom is defined as an learning ecosystem where human agents (teachers and students) and intelligent computational agents (AI-based tutoring systems, adaptive platforms, and natural language processing tools) engage in bidirectional, goal-oriented interaction to co-construct linguistic knowledge (Dai et al., 2024; Boubker, 2024). Unlike traditional computer-assisted language learning (CALL), where technology functions as a passive tool, the computer in this model acts as an active partner with agency—providing real-time feedback, negotiating meaning, and adapting to learner input (Memarian & Doleck, 2024). This collaboration occurs across three planes: (a) student-computer dyads (one-on-one AI tutoring), (b) teacher–computer orchestration (AI-informed instructional design), and (c) student–student–AI triads (peer collaboration mediated by shared AI resources). Human–computer collaboration enhances second language cognitive engagement by allowing students to offload lower-order tasks (e.g., vocabulary recall) to AI, freeing cognitive resources for higher-order thinking (e.g., pragmatic appropriateness) (H. Ji et al., 2022). Simultaneously, AI’s metacognitive prompts (“Why did you choose this tense?”) push students to externalize reasoning, deepening linguistic analysis.

### 2.2. Student Engagement in Human–Computer Collaborative Classroom

Previous studies indicate that human–computer collaborative learning methods, including group projects, peer feedback, and cooperative tasks, significantly enhance student motivation and academic performance (Ramadan Elbaioumi Shaddad & Jember, 2024). Traditional learning environments often emphasize individual engagement, such as personal focus and time investment. However, in collaborative learning contexts, it is crucial to shift the emphasis from individual to group cognition. Group cognition encompasses shared knowledge construction, intellectual exchanges, and the collaborative development of problem-solving strategies among group members, which effectively demonstrate students’ deep engagement and interaction in collaborative learning (Z. Guo et al., 2023). Recent studies have increasingly focused on English teachers’ views regarding English as a Foreign Language (EFL) students’ engagement in collaborative learning, examining its implementation, benefits, and challenges from the teachers’ perspective (Chan et al., 2022). Teachers are pivotal in this environment, enhancing student experiences through task design and support (Lustenberger, 2023). While these studies shed light on language acquisition in collaborative settings, research on the engagement of Mongolian-speaking students learning Chinese through human–machine collaborative teaching remains limited.

### 2.3. Assessment for Student Engagement in Human–Computer Collaborative Classroom

Learning engagement, a crucial metric for assessing student participation and outcomes (Ni & Wu, 2011), encompasses the positive attitude, intense focus, and persistent mindset students exhibit during learning activities. Research indicates that learning engagement is context-dependent, with its conceptual nuances varying across different settings. These variations include aspects such as learning motivation (Wun-Cin et al., 2024), learning gains, active learning, attitude, time investment, methods (Lu, 2017), strategies (Mizumoto & Chujo, 2016), and the teaching environment.

Assessment of student engagement in human–machine collaborative teaching primarily involves behavioral data analysis, questionnaires, interviews, and classroom observation. Behavioral data analysis evaluates engagement by examining metrics such as learning duration, frequency, clicks, and answer accuracy within computer-assisted learning systems. Techniques like deep learning models can assess students’ behavioral and emotional traits to gauge engagement (Mahmood et al., 2024). Questionnaires capture cognitive, emotional, and behavioral responses to the teaching model, offering insights into engagement levels. These tools may include components like emotional dimensions and attitudes, as seen in assessments for Indonesian language learning (Pani & Handayani, 2024). Interviews provide in-depth insights into students’ experiences, emotions, and challenges, facilitating a comprehensive evaluation of engagement (Tong et al., 2025). Classroom observation involves monitoring student behaviors, such as participation in discussions, task completion, and peer interaction, to assess engagement (Zhu & Yang, 2024).

### 2.4. Dimensions of Student Engagement in Human–Machine Collaborative Classroom

Ethnic minority students in China often struggle with Mandarin language acquisition due to limited exposure and restricted access to academic language during formative years, resulting in proficiency gaps compared to Han nationality peers. Although some excel in social Mandarin communication, they face challenges in academic settings (H. Wang & Chao, 2021). For these students, classroom Mandarin learning involves second language acquisition and mastering complex academic language. Unlike everyday language, academic language is characterized by lexical diversity, discourse abstraction, and syntactic complexity (Liang et al., 2021), which ethnic minority students do not naturally acquire in daily life.

The Mandarin classroom is pivotal in supporting academic, social, and learning activities for ethnic minority students, aiding their integration into a multilingual and multicultural society and enriching their learning experience. As an essential platform for researching Mandarin learning engagement, the intelligent classroom effectively integrates information technology into educational processes. This integration has made Mandarin learning ubiquitous among ethnic minority secondary school students, emphasizing literacy and cultural responsiveness (X. Huang et al., 2021). Considering the complex nature of learning engagement and the specific characteristics of Mandarin classroom learning for these students, this study categorizes critical indicators of learning engagement into seven dimensions: higher-order thinking, student–teacher interaction, peer interaction, human–computer interaction, active learning, learning challenges, and enthusiasm for learning Mandarin.

Higher-order thinking skills (HOTS) are crucial predictors of academic and professional success (Y.-M. Huang et al., 2022). HOTS encompass cognitive processes like analysis, evaluation, and creation (Sulistyorini et al., 2020), extending beyond mere memorization and comprehension. These skills necessitate critical thinking, problem-solving, and decision-making. Classroom engagement, characterized by active participation, interest, and concentration, directly influences learning outcomes and knowledge acquisition (J. Wang et al., 2022a). Theoretically, higher-order thinking skills (HOTS) can enhance students’ intrinsic motivation and engagement. By employing analytical and evaluative skills, students are more likely to grasp the deeper meanings of knowledge, thereby boosting their interest and enthusiasm for learning. In collaborative settings, interaction with AI systems offers personalized learning experiences and immediate feedback, further increasing their engagement (Yatani et al., 2024).

Teacher–student interaction is fundamental to classroom teaching, significantly influencing students’ interest, motivation, and engagement. Through questioning, discussion, and feedback, teachers encourage active participation, critical thinking, and problem exploration. This interaction not only aids in knowledge comprehension but also fosters a sense of belonging and achievement, thereby enhancing engagement (Wei, 2021; Mehmood & Taresh, 2024). For students learning the national language in Mongolian, such interaction is crucial in overcoming cultural and linguistic barriers, boosting their confidence. Bibliometric analyses further reveal that in English language teaching, student participation is closely linked to teacher–student interaction (Hınız & Çelik, 2024).

Sociocultural theory underscores the significance of social interaction in learning. Vygotsky argued that learning is not an isolated endeavor but a collaborative process of knowledge construction within a sociocultural context. In the context of Mandarin acquisition, Mongolian students benefit from peer interactions, receiving immediate feedback, correcting pronunciation, and acquiring authentic expressions, which boosts their confidence and enthusiasm for language learning (S. Guo & Möllering, 2016). Additionally, peer interaction offers a platform for students to demonstrate language skills and articulate their views, facilitating language internalization and application. Research shows that effective peer interaction markedly enhances students’ oral fluency and accuracy, thereby increasing overall learning engagement (Campbell et al., 2024).

According to social cognitive theory, students’ learning engagement is shaped by factors such as self-efficacy, motivation, and the external environment (Mekheimer, 2025). Human–computer interaction, an external factor, can modulate engagement by affecting self-efficacy and motivation. Gamified learning, immediate feedback, and tailored exercises, for instance, can enhance students’ interest and intrinsic motivation (Xiong et al., 2022). When learning content and interaction methods align with students’ needs and preferences, they are more inclined to dedicate time and effort to learning (Trubitzina et al., 2022).

Cognitive engagement theory posits that students actively construct knowledge during learning. This requires them to think critically, ask questions, engage in collaborative exploration, and reflectively summarize. Such activities enhance students’ deep understanding and internalization of knowledge (Narciss et al., 2022). For native Mongolian speakers, learning Mandarin presents additional linguistic and cultural adaptation challenges. Human–machine collaborative teaching can address these issues by offering personalized language resources and interactive exercises (J. Li et al., 2023), thereby fostering active learning behaviors.

Learning challenges significantly enhance student motivation and engagement. When faced with tasks of suitable difficulty, students are more likely to allocate cognitive resources and exert effort to find solutions (Ginting, 2021). These challenges activate learning potential, encouraging active exploration and deeper understanding of the material (Y. Wang & Stein, 2021). In Mandarin classes, this deep learning is evident in increased questioning, discussion participation, and interaction with teachers and peers, leading to improved learning outcomes (Peeters et al., 2020).

In Mandarin classes, active student engagement is crucial due to the interactive nature of language learning (C. Ji et al., 2017). Learning enthusiasm, an internal motivator, significantly affects this participation (Uyun et al., 2022). Within the framework of positive psychology, elements like positive emotions, engagement, interpersonal relationships, meaning, and achievement constitute well-being (Gregersen, 2019). Enthusiasm for learning Mandarin can be seen as a positive emotion. Students with high enthusiasm are more inclined to collaborate with peers and tackle problems collectively (Harper & Chen, 2018; Sedláček & Šeďova, 2020), thereby enhancing their participation in classroom activities. This engagement fosters a deeper involvement in the learning process, ultimately leading to a sense of achievement.

Therefore, this study is conducted around the following research issues:

**RQ1.**  *What are the core dimensions of learning engagement experienced by ethnic minority students in human–computer collaborative classrooms?*

**RQ2.**  *What is the factorial structure and psychometric quality (reliability, validity) of h* *uman–computer collaborative classroom second language learning engagement scale?*

## 3. Methodology

### 3.1. Participants

China’s implementation of the Minority Autonomous Regions and ethnic regional autonomy system provides a suitable context for investigating minority students, making them valuable subjects for this study. In terms of participant selection, stratified sampling is deemed appropriate as it allows for the acquisition of samples that possess similar characteristics to the total population while simplifying the sampling process and facilitating operational efficiency. Separate samples were recruited for EFA and CFA to avoid data-driven bias and enhance cross-validation robustness. For the purpose of this study, a total of 710 participants were selected from seven secondary schools located in the Chinese Minority Autonomous Regions. These participants commenced their Mandarin curriculum in the third grade and have accumulated over three years of experience in second language acquisition.

Exploratory factor analysis selected 437 valid samples, among which 189 were male participants (43.2%) and 248 were female participants (56.8%). Regarding the participants’ place of origin, 91 samples were from urban areas (21%), 105 from townships (24%), and 241 from rural pastoral areas (55%). Confirmatory factor analysis selected a separate set of subject data (N = 273). Regarding gender differences, the sample consisted of 140 male students, accounting for 51.3%, and 133 female students, accounting for 48.7%. In terms of their place of origin, 58 ethnic minority secondary school students hailed from urban areas (21.2%), 89 from townships (32.6%), and 126 from rural herding areas (46.2%).

To gather data, a stratified random sampling method was employed, incorporating web-based and field surveys to conduct interviews with ethnic minority students.

### 3.2. Instrument

In this study, a English version of the mathematics and science learning engagement scale was developed by M. T. Wang et al. (2016) based on the National Survey of Student Engagement scale (Fuller et al., 2011), with additional references from other scales. The scale consisted of two parts. The first part encompassed basic personal information, featuring seven items covering gender, ethnicity, place of origin, school, sibling status, and parents’ education level. The second part consisted of a survey focusing on learning engagement in the Mandarin classroom among ethnic minority students in secondary schools. This section encompassed various dimensions, including higher-order thinking (metacognitive strategies and cognitive strategies; 13 items), student–teacher interaction, peer interaction, and human–computer interaction, learning challenges, and active learning (14 items), as well as self-efficacy and identity (9 items), resulting in a total of 60 items. Among them, the items of higher-order thinking, human–computer interaction and enthusiasm for Chinese language learning were developed based on interview codes; the items of student–teacher interaction, peer interaction, learning challenges and active learning were adapted from the NSSE questionnaire in the United States. The specific composition of each dimension is as follows: higher-order thinking includes cognitive strategies (3 items, A1–3) and metacognitive strategies (4 items, B1–4); student–teacher interaction refers to the interaction between minority students and teachers (3 items, C1–3); peer interaction refers to the interaction among minority students (3 items, D1–3); human–computer interaction refers to the interaction between minority students and computers/platforms/multimedia (3 items, E1–3); learning challenges refer to academic challenges (4 items, F1–4); active learning refers to the active performance of minority students in the Chinese language classroom (6 items, G1–6); enthusiasm for Chinese language learning encompasses interest in Chinese language learning (6 items, H1–6) and Chinese language learning identity (5 items, I1–5).

The survey aimed to measure the level of engagement of ethnic minority students in Mandarin classroom learning. Each item was measured using a 7-point Likert scale, ranging from 1 for “strongly disagree” to 7 for “strongly agree.” All items were stated affirmatively, and it is important to note that several reverse-scored items from the original scale, such as “In the Mandarin classroom, I do not participate” and “If I do not understand, I will drop it immediately,” were excluded (see in Table 1).

It should be noted that this scale does not include reverse-coded items. Although reverse-coded items have potential value in mitigating implicit biases, second language (L2) learners (especially intermediate-level learners) are prone to misunderstandings when understanding and responding to reverse statements. And cross-cultural measurement studies have shown that when learners have limited language proficiency, reverse-coded items reduce measurement invariance and thereby increase systematic errors (Boer et al., 2018).

To ensure linguistic equivalence and semantic fidelity during the translation-back-translation process, two independent bilingual experts (PhD in Applied Linguistics, >10 years L2 teaching experience) conducted forward translation separately. A third expert (native English speaker, professional translator) blindly back-translated the Chinese version into English without viewing the original items. Subsequently, a panel of four experts (including the first two translators and two additional TESOL researchers) independently coded item-level semantic congruence on a 3-point scale (1 = not equivalent; 2 = somewhat equivalent; 3 = fully equivalent). Intercoder reliability was assessed using Cohen’s kappa for pairwise agreement. The pairwise Cohen’s kappa ranged from 0.78 to 0.92 (all *p* < 0.001), indicating substantial to almost perfect agreement. Discrepancies were resolved through consensus discussion, and items with initial kappa < 0.70 were reworded based on panel feedback until unanimous agreement was achieved.

### 3.3. Research Procedure and Data Collection

This study was conducted over an 8-month period across four stages. Each stage involved specific participant–tool interactions (see in Table 2).

This study employed a sequential mixed-methods design, integrating qualitative item generation and quantitative psychometric validation in two distinct phases. First, Semi-structured interviews and focus groups were conducted to generate an initial item pool and ensure content validity within the ethnic minority CFL context. Secondly, A survey was administered for exploratory factor analysis (EFA) and confirmatory factor analysis (CFA), followed by criterion validity testing.

This study aims to develop a model for explaining and predicting learning engagement in the Mandarin classroom for ethnic minority students in secondary schools. The data analyzed in this study were obtained from a questionnaire survey; thus, IBM SPSS Statistics 26.0 and IBM AMOS 24.0 software were used to validate the scale. The validation process involved item analysis, exploratory factor analysis, confirmatory factor analysis, and reliability analysis. The development of the scale occurred in three stages.

In the initial stage, semi-structured interviews and open-ended questionnaires were administered to gather insights from ethnic minority students. The findings from these interviews, combined with a thorough review of the literature, were utilized to design the dimensions and specific scale items for learning engagement in the Mandarin classroom, resulting in the creation of a preliminary questionnaire.

Subsequently, exploratory factor analysis was conducted on the data obtained from the preliminary scale, leading to the removal and consolidation of dimensions and items. This process yielded a refined scale of learning engagement in the Mandarin classroom among ethnic minority students. Exploratory factor analysis was further employed to analyze the data from the preliminary questionnaire, enabling the reduction in scale factors and items and ultimately culminating in the construction of a standardized scale for assessing learning engagement in the Mandarin classroom among ethnic minority students in secondary schools.

Finally, confirmatory factor analysis was employed to validate the scale’s validity, ensuring its suitability and reliability for assessing learning engagement in the Mandarin classroom among ethnic minority students.

## 4. Data Analysis and Results

### 4.1. Item Analysis

Initially, the total scores based on 37 items were computed for each participant, followed by a correlation analysis between individual item scores and the total score. The results revealed significant correlations between the scores of all 37 items and the total score. This indicates a high level of homogeneity between each item and the overall scale, thereby validating their suitability for subsequent statistical analyses.

The scores of each student on all items were aggregated and arranged in descending order. Subsequently, considering the total sample size (N = 437), a critical value of 215 was established for the top 27% of the high subgroup, while a critical value of 171 was determined for the bottom 27% of the low subgroup. Participants with a total score equal to or exceeding 215 were classified as the high group, while those with a total score equal to or below 171 were classified as the low group. Following this categorization, independent sample *t*-tests were conducted to examine the between-group differences for each item. The findings revealed significant differences (*p* < 0.001) between the high and low groups across all items, indicating that all 37 items successfully passed the critical ratio analysis, exhibited adequate discrimination, and did not require removal.

The homogeneity of variance test method, comprising reliability analysis, communality, and factor loadings, serves as a crucial tool for effective item screening. In this study, principal component analysis (PCA) was employed to analyze common factors. The initial standard value was set at 1, with a reference extracted value of 0.2. Results indicated that the communality of item F3 was 0.132, falling below the threshold of 0.2. This low communality suggested inadequate association between item F3 and the overall scale. Additionally, cronbach’s α was utilized to assess the scale’s reliability. The total scale demonstrated a cronbach’s α value of 0.959 and McDonald’s ω value of 0.966. However, when item F3 was deleted, the cronbach’s α if item deleted increased to 0.961, surpassing the initial value of 0.959. This finding suggests that the internal structural consistency of the scale improved after removing item F3.

In conclusion, item F3 should be excluded due to its failure to meet the criteria for corrected item-total correlation (CITC), cronbach’s α if item deleted, communality, and factor loadings. Conversely, the remaining items demonstrated values within an acceptable range and should be retained.

### 4.2. Exploratory Factor Analysis

To assess the suitability of the sample data for factor analysis, the Kaiser–Meyer–Olkin (KMO) measure and Bartlett’s test of sphericity were computed. As depicted in Table 3, the KMO value of 0.918 substantially exceeds the conventional threshold of 0.7. This high KMO coefficient suggests that the items share considerable common variance, which affirms the theoretical premise that cognitive, emotional, and behavioral engagement dimensions are interrelated manifestations of an underlying second language learning engagement construct rather than independent phenomena. Furthermore, Bartlett’s test of sphericity yielded an approximate chi-square value of 4002.369 (df = 171, *p* < 0.001), rejecting the null hypothesis that the correlation matrix is an identity matrix. This result provides evidence that the observed item intercorrelations are not attributable to sampling error alone but reflect patterned relationships suitable for factor extraction. For our engagement scale, this patterning is theoretically expected: the latent structure is predicated on the assumption that human–computer collaborative L2 learning generates synergistic interactions among engagement dimensions. Bartlett’s result substantiates that our data exhibit the necessary non-random covariance structure to reveal such latent factors, thereby supporting the construct validity of our measurement model.

**Figure 1:**
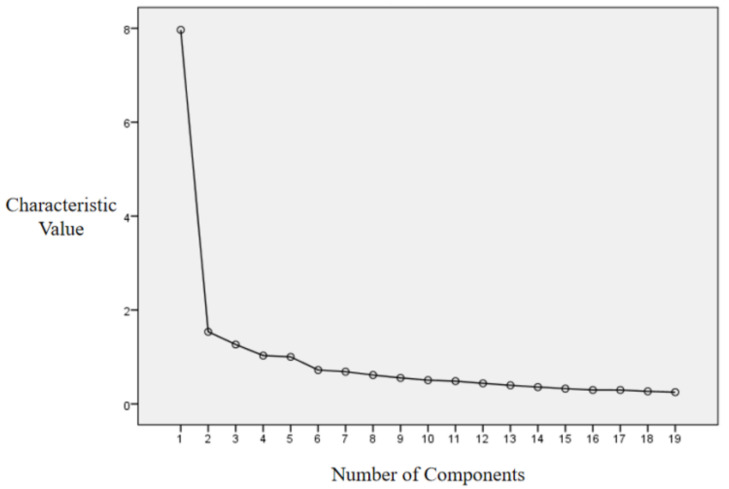
Gravel Plot of ICT-supported Factor Analysis of Student Engagement in Chinese Language Classroom.

In this study, multiple exploratory factor analyses were performed iteratively by excluding individual items. If the factor loading of a certain item was below the threshold of 0.60, it would be excluded. Following this iterative process, a total of 18 items were removing, leaving 19 items. Five factors with eigenvalues greater than 1 were extracted, accounting for a cumulative variance of 67.372%. The factor analysis gravel plot for learning engagement in Chinese language classrooms in an ICT environment (Figure 1) indicated that extracting 4–6 factors was appropriate. The rotated factor loading matrix (N = 437) in Table 4 (refer to the Appendix A for the scale items) revealed that extracting 5 factors was the most suitable choice.

The rotated factor loading matrix reveals that ethnic minority students’ engagement in Chinese language classroom learning can be categorized into five factors. The first factor comprises four items: I1, I3, I5, and H4. These items gauge the interest and identification of ethnic minority students with Chinese language classroom learning. The second factor consists of five items: A1, A2, B1, B2, and B3. These items primarily capture the application of metacognitive and cognitive strategies employed by ethnic minority students in secondary schools, indicating a focus on “higher-order thinking.” The third factor encompasses four items: D1, D2, G3, and G4. These items describe the active learning and peer interactions of ethnic minority students in Chinese language learning, thus aptly termed “active collaborative learning.” The fourth factor comprises three items: E1, E2, and E3, which assess the level of human–computer interaction in Chinese language classrooms among ethnic minority students in secondary schools, justifying the label “human–computer interaction.” Lastly, the fifth factor includes three items: C1, C2, and C3, which primarily explore the interaction between ethnic minority students and teachers, encapsulating the concept of “student–teacher interaction.”

Following the aforementioned process of exploratory factor analysis, the “Human–Computer Collaborative Classroom Second Language Learning Engagement Scale (Official Version)” was successfully developed. The scale comprises a total of 19 items, distributed across five factors: higher-order thinking (5 items), student–teacher interaction (3 items), human–computer interaction (3 items), active collaborative learning (4 items), and enthusiasm for Chinese language learning (4 items). The scale adopts a 7-point Likert scale, with participants providing self-reported declarative statements. All items are phrased positively, and higher total scores indicate greater levels of engagement in Chinese language classroom learning among ethnic minority students in secondary schools. Conversely, lower total scores reflect lower levels of learning engagement in Chinese language classrooms among ethnic minority students in secondary schools.

### 4.3. Confirmatory Factor Analysis

In this study, two indicators, cronbach’s α and composite reliability (CR) were used to test the internal consistency of the scale of learning engagement in Chinese language classroom among ethnic minority Students (see Table 5). The CR value is generally considered favorable when greater than 0.7.

The statistical analysis results in Table 4 show that the cronbach’s α for the dimensions of higher-order thinking, student–teacher interaction, active collaborative learning, human–computer interaction, and enthusiasm for learning the Chinese language in the scale are 0.888, 0.842, 0.873, 0.849, and 0.867, respectively. These values exceed 0.8, denoting a high level of internal consistency across the factors of learning engagement in the Chinese language classroom. Moreover, as displayed in Table 4, the reliability values for all factors of learning engagement in the Chinese language classroom exceed 0.8, meeting the requirements of reliability analysis. Thus, it can be concluded that the scale demonstrates a high level of reliability.

Validity analysis encompasses content and construct validity. To ensure the content validity of the “Human–Computer Collaborative Classroom Second Language Learning Engagement Scale,” five education professors and ten ethnic minority students were invited to evaluate the scale’s dimension formulation and question design. Any uncertainties were thoroughly discussed and appropriately revised. Based on the feedback received, the scale was deemed well-designed and capable of effectively capturing the engagement of ethnic minority students in Chinese language learning within secondary schools. Hence, the scale exhibits satisfactory content validity.

Structural validity examines whether the measurement questions accurately and reliably reflect the structural dimensions of ethnic minority students’ engagement in Chinese language classrooms, specifically encompassing five factors: higher-order thinking, student–teacher interaction, human–computer interaction, active and cooperative learning, and enthusiasm for Chinese language learning. In this study, structural validity was assessed through convergent validity and discriminant validity. Convergent validity, as demonstrated in Table 6, evaluated the similarity of results obtained from different measures of classroom learning engagement. The results revealed that all items of the scale exhibited factor loadings exceeding 0.7. The average variance extracted (AVE) exceeded 0.6 for all factors, and the composite reliability (CR) exceeded 0.8, meeting the criteria for sound judgment. These findings indicate that the scale possesses acceptable convergent validity.

A discriminant validity analysis was conducted to examine the degree of difference between the factors of higher-order thinking, student–teacher interaction, human–computer interaction, active collaborative learning, and enthusiasm for Chinese language learning. If there is no significant difference between all the variables in the model, the discriminant validity of model is satisfactory. AMOS 24.0 was used to conduct confirmatory factor analysis of each factor in different combinations in this study. In this regard, the model fitting indexes are the degree between the data and the constructed model, and the specific fit parameters are shown in Table 7.

**Figure 2:**
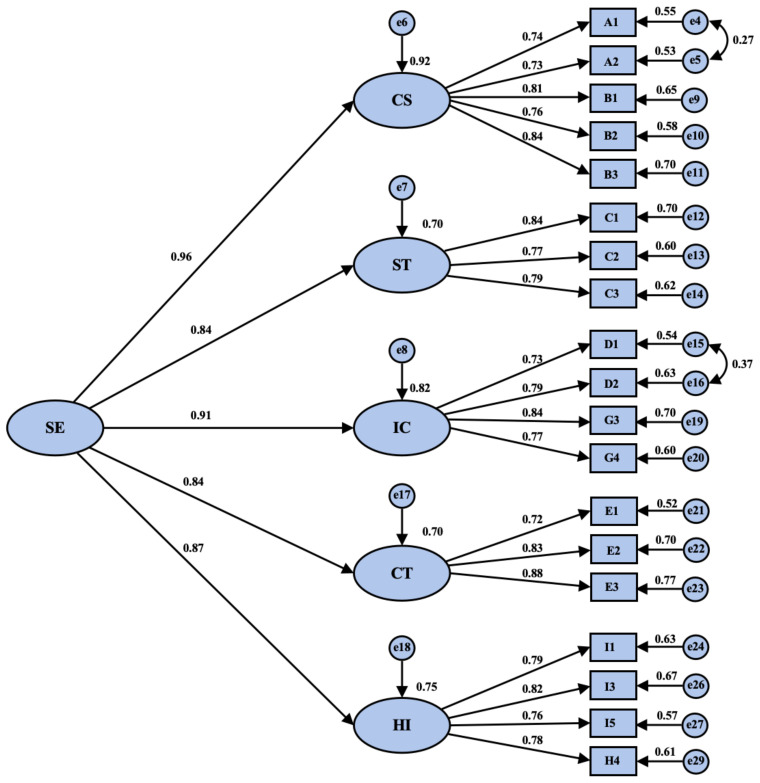
Parameter estimates for the confirmatory factor analysis of the five-factor model. Note. CS stands for higher-order thinking, ST stands for student–teacher interaction, IC stands for active collaborative learning, CT stands for human–computer interaction, and HI stands for enthusiasm for Chinese classroom learning.

Upon comparing the fit indices of the four-factor model, three-factor model, two-factor model, and one-factor model, the five-factor model (comprising higher-order thinking, student–teacher interaction, active collaborative learning, human–computer interaction, and enthusiasm for Chinese language learning) demonstrated the best fit. In comparison to alternative factorial models, the χ^2^/df value of the five-factor model was 1.457, falling within the range of 1 to 3 and satisfying the goodness-of-fit criterion. The root mean square error of approximation (RMSEA) was 0.041, significantly below 0.08 and approaching zero, indicating an excellent fit to the model. Moreover, the normative fit index (NFI) was 0.941, the comparative fit index (CFI) was 0.981, the goodness-of-fit index (GFI) was 0.925, the adjusted goodness-of-fit index (AGFI) was 0.902, the Tucker–Lewis index (TLI) was 0.977, and the incremental fit index (IFI) was 0.981. All five indices exceeded the critical value of 0.9. These values indicate a favorable fit of the factor data to the model, confirming that the five-factor model possesses strong discriminant validity. Furthermore, the confirmatory factor analysis outcomes of the five-factor model are displayed in Figure 2.

## 5. Conclusions and Discussion

This study introduces a analytical model to evaluate Second Language Learning Engagement in Human–Computer Collaborative Classroom. The model encompasses five key dimensions: higher-order thinking, student–teacher interaction, human–computer interaction, active collaborative learning, and enthusiasm for learning Chinese. By defining these dimensions and conducting empirical analysis, the study establishes a valid and reliable measurement scale. This scale is a crucial tool for educational researchers to assess learning engagement and supports cross-sectional research on instructional interventions like online collaborative writing. Initial validation and reliability testing may have been limited by sample size.

### 5.1. Validation of Human–Computer Collaborative Classroom Second Language Learning Engagement Scale

The study introduced the “Human–Computer Collaborative Classroom Second Language Learning Engagement Scale” through a meticulous process that involved deriving dimensions and items from extensive research, analysis of interview coding outcomes, and consultation with subject matter experts. The research outcomes underscore the significant influence of various factors such as higher-order thinking, student–teacher interaction, human–computer interaction, active collaborative learning, and enthusiasm for learning the Chinese language on students’ engagement within the Chinese language classroom.

Through exploratory factor analysis, the study identified five factors with eigenvalues greater than one, with all items demonstrating substantial factor loadings exceeding 0.60. These factors collectively explained 67.372% of the total variance. Subsequent confirmatory factor analysis (CFA) provided further validation for the five-factor model, with all indicators surpassing the established thresholds. This outcome reinforced the discriminant validity initially observed in the exploratory factor analysis. Moreover, the scale and its constituent factors exhibited strong reliability, as indicated by Cronbach’s α coefficients exceeding 0.80, signifying robust internal consistency among the factors and high homogeneity among the scale items. These assessments collectively affirm the scale’s robust reliability and validity.

It is worth noting that this study reduced the initial seven hypothesized variables to five empirically validated factors, and this adjustment was driven by statistical evidence and the principle of conceptual simplification. Firstly, at the statistical level, the items related to peer interaction had a high cross-load on the active learning factor (>0.40), so some of these items were incorporated into the active learning factor; Confirmatory Factor Analysis (CFA) further revealed that the stability of the learning challenge-related items was poor, so they were removed. Secondly, at the conceptual and theoretical level, the final model did not consider peer interaction as an independent factor, but embedded it in the active collaborative learning dimension, which is in line with Vygotsky’s social constructivist theory (Payong, 2020). The removal of the learning challenge factor was consistent with the Self-Determination Theory (SDT). Challenges are not direct motivating factors, but rather a moderating variable that expands participation when there is support for abilities (Ryan & Deci, 2020). Qualitative data also indicated that the learning challenge in the human–computer interaction (HCI) environment was perceived as a variable that “enhances abilities” or “destroys autonomy”, and thus only existed as a background condition rather than a stable participation dimension.

### 5.2. Reconceptualizing Engagement in Human–Computer Collaborative L2 Chinese Classrooms for Ethnic Minority Students

The primary aim of this study was to develop and validate a context-specific scale for assessing second language (L2) learning engagement among ethnic minority secondary school students in human–computer collaborative Chinese classrooms. Five dimensions—higher-order thinking, student–teacher interaction, human–computer interaction, active collaborative learning, and enthusiasm for Chinese language learning—emerged as core constituents of engagement. Among these, “higher-order thinking” emerged as pivotal in enhancing learning engagement in Chinese language classrooms for ethnic minority students, reflecting their ability to comprehend Chinese at an advanced cognitive level. The findings indicate that higher-order thinking is not merely an isolated cognitive strategy but a comprehensive process encompassing both metacognitive and cognitive strategies. It involves effectively monitoring and controlling cognitive processes and outcomes, alongside employing diverse thinking methods or skills to achieve cognitive objectives. Therefore, Chinese language teachers should guide students in developing self-evaluation skills and methods to identify existing challenges, thereby fostering higher-order thinking in ethnic minority secondary school students.

In intelligent education, the integration of human and machine interactions has blurred the lines between physical space, information space, and human society. This shift has redefined interactions in the Chinese language classroom into “human–machine interaction,” “student–teacher interaction,” and “active collaborative learning.” Human–computer interaction involves communication between ethnic minority students, teachers, and intelligent devices like PCs or tablets. Through digital interfaces, teachers can track students’ progress, quizzes, and online activity. Students receive personalized guidance, feedback, and digital resources for Chinese language content. These interactions between students and teachers significantly enhance classroom engagement. In a blended learning environment that merges physical and digital spaces, the dynamic interaction between ethnic minority students and teachers effectively connects the interactions between humans and machines, humans and resources, and humans and learning spaces. It redefines a bilateral activity marked by rich teaching experiences and a strong sense of social presence. “Active collaborative learning” describes the dynamic and positive behaviors of ethnic minority students in Chinese language classrooms. In blended learning environments, teachers can use strategies like collaborative group learning, brainstorming, and concept or mind mapping to restructure the classroom, thereby stimulating active learning among these students.

Students’ emotions in Chinese language learning are shaped by the classroom environment and their attitudes toward the language. “Enthusiasm for Chinese language learning” refers to the psychological traits of ethnic minority secondary school students who are inclined to appreciate, study, and accept Chinese language knowledge. This enthusiasm combines interest in learning Chinese with a sense of identity. Fostering and sustaining this interest among ethnic minority students is crucial for boosting classroom engagement in Chinese language instruction, as it forms the foundation for their learning enthusiasm.

Compared with earlier engagement instruments developed for generic L2 contexts (e.g., Fredricks et al., 2004), the present scale foregrounds technology-mediated, culturally situated facets of engagement. While previous studies treat human–computer interaction as a peripheral component of behavioral engagement (Alkatheiri, 2022), our qualitative vignettes and CFA results reveal that intelligent devices function as co-agents that afford real-time diagnostic feedback, adaptive scaffolding, and identity-safe spaces for ethnic minority learners.

Theoretically, the salience of enthusiasm for Chinese language learning underscores the affective–identity nexus in L2 engagement, a dimension often under-specified in mainstream engagement frameworks (Chen et al., 2024). Our data indicate that ethnic minority students’ willingness to invest in Chinese is contingent upon the perception that the language is compatible with, rather than erosive of, their ethnic identity. Furthermore, in the human–computer collaborative teaching for minority students, we fully recognize that the cultural background (especially the sociolinguistic background of minority students) fundamentally regulates the learners’ perception and interaction methods with digital tools. learning engagement is not a culturally neutral concept; its essence is mediated by culture and is rooted in the negotiation process of students’ language history, digital capital, and cultural identity. Practically, these findings suggest that culturally responsive, technology-enhanced pedagogies should explicitly legitimize students’ multilingual repertoires while leveraging AI analytics to visualize incremental gains, thereby sustaining enthusiasm.

### 5.3. Comparative Explanatory Power: Positioning Against Established Scales

The integration of technology into second language (L2) learning environments has catalyzed a paradigm shift in how learner engagement is conceptualized, measured, and fostered. We therefore position our Human–Computer Collaborative L2 Learning Engagement Scale against three benchmarks: (1) the Student Engagement Instrument (SEI; Appleton et al., 2006), representing general academic engagement; (2) L2 engagement framework (J. Wang et al., 2022b), representing domain-specific but technology-generic engagement; and (3) the Online Learning Engagement Scale (OLES; Tsai et al., 2021), representing broader technology-enhanced learning. However, these benchmarks—while robust within their respective scopes—do not fully account for the dynamic, bidirectional interactions that characterize human–AI co-agency, a phenomenon increasingly central to contemporary L2 learning ecosystems.

The SEI operationalizes engagement through cognitive and psychological dimensions, emphasizing students’ investment in school-related tasks and their affective connection to educational settings. It captures key motivational constructs such as self-efficacy, goal orientation, and perceived competence, which are critical predictors of academic persistence. Yet, its design predates the widespread adoption of generative AI tools like ChatGPT or intelligent tutoring systems, rendering it less sensitive to real-time, adaptive feedback loops between learners and AI agents. Similarly, L2 engagement framework advances our understanding of language-specific engagement by incorporating sociocognitive and affective variables such as task enjoyment, willingness to communicate, and identity negotiation. While this model provides granular insights into language learning motivation, it does not explicitly address how AI-mediated scaffolding—such as automated grammar correction, personalized vocabulary drills, or conversational simulation—alters the trajectory of engagement over time.

In contrast, the OLES represents a significant step toward measuring engagement in digital learning spaces, focusing on interactivity, autonomy, and instructor presence in online courses. García-Machado et al.’s (2024) scale acknowledges technological affordances but remains anchored in human–human interaction paradigms, treating technology primarily as a conduit for teacher–student or peer communication rather than as an active agent in the learning process. This limitation becomes particularly salient when examining AI-driven environments where the machine itself assumes pedagogical roles—offering explanations, prompting reflection, and even modeling linguistic behavior.

Thus, the explanatory power of the Human–Computer Collaborative L2 Learning Engagement Scale lies precisely in its capacity to move beyond redundancy and illuminate phenomena invisible to prior frameworks. By anchoring measurement in co-regulated learning processes, joint problem-solving episodes, and mutual adaptation patterns, this scale offers a theoretically grounded and psychometrically rigorous means of assessing how learners and AI systems collaboratively construct meaning, negotiate understanding, and co-evolve over time.

### 5.4. Implications for Assessing and Enhancing the Quality of Ethnic Minority Education in the Intelligent Era

The education sector is undergoing a transformation driven by advanced information technologies such as 5G, big data, and artificial intelligence. This shift towards intelligent education is reshaping classroom practices through smart learning environments, integrated applications, and tailored teaching services (Zhai et al., 2021). In this evolving landscape, Chinese language instruction for ethnic minority secondary students has become increasingly complex. As Chinese language learning is categorized under second language acquisition (D. Li & Ouyang, 2021), examining engagement in these classrooms requires a multidisciplinary approach, integrating insights from linguistics, education, psychology, sociology, and data science. Thus, a systematic study of student engagement in Chinese language classes for ethnic minorities is essential for evaluating the quality of ethnic education and informing effective strategies.

The Chinese language classroom is crucial in evaluating national education quality and individual education. Despite transitions from “digital” to “intelligent” learning environments and the diversification of theoretical frameworks for Chinese language learning, the focus on learning engagement remains unwavering, adhering to the core educational purpose. This scale targets dimensions such as “higher-order thinking,” “student–teacher interaction,” “human–computer interaction,” and “active collaborative learning,” offering a comprehensive research perspective and profound implications for individual development. Our findings intimate that learning engagement is a culturally mediated, multidimensional construct that cannot be reduced to behavioral attendance or test scores.

Compared with smart-classroom evaluation index, which privileges infrastructure indicators (bandwidth, device ratio), our scale shifts the analytical gaze to learner-centered processes. This reorientation resonates with Peng et al.’s (2021) argument that effectiveness of Chinese L2 instruction for minorities hinges upon sustained cognitive–affective involvement rather than mere access to digital tools. It highlights the importance of learning engagement in fostering the growth of ethnic minority secondary school students. Intelligent education must prioritize the personal growth of ethnic students, adapting to changes in ethnic education, creating environments conducive to their development in both physical and digital spaces, and ultimately providing support for the information technology revolution.

## 6. Limitations and Future Directions

In fact: the cross-sectional validation design of this study does not support strong causal inferences. Future research could establish causal pathways and specific predictive relationships between the five factors and learning engagement, facilitating a shift in discussions from mere correlational explanations to mechanism-driven theoretical construction. It is also recommended that future studies adopt multilevel longitudinal designs, triangulating trace data from learning management systems with experience-sampling affect logs to capture the microgenesis of engagement. Additionally, long-term cohort studies are needed to explore whether early engagement profiles predict subsequent learning engagement and language vitality. Integrating large-scale psychometric modeling with ethnographic case studies will generate mixed-evidence dashboards that can inform minority education policies. 

## Figures and Tables

**Table 1:** Overall Structure and Content of Human–Computer Collaborative Classroom Second Language Learning Engagement Scale.

Factors	Indicators and Codes	Items	Source	Scoring
Higher-Order Thinking	Cognitive strategies (A1–3), metacognitive strategies (B1–4)	7	Interview Codes	7-point Likert
Student–Teacher Interaction	Interaction between ethnic students and teacher (C1–3)	3	NSSE	
Peer Interaction	ethnic student to student interaction (D1–3)	3	NSSE	
Human–Computer Interaction	Ethnic students and the PC/platform/multimedia Interaction (E1–3)	3	Interview Codes	
Learning Challenges	Academic challenges (F1–4)	4	NSSE	
Active Learning	Positive response of ethnic students in Chinese language classroom (G1–6)	6	NSSE	
Enthusiasm for Chinese Language Learning	Interest in Chinese language learning (H1–6), sense of identity in Chinese language learning (I1–5)	11	Interview Codes	

**Table 2:** Participant–Tool Interaction Details.

Stage	Time Period	Participant Actions	Researcher Actions	Tool Interaction Mode
1. Item Generation	2 months	-Signed informed consent -Completed demographic form (5 min) -Participated in 1-on-1 interview (45–60 min) or focus group (90 min)	-Administered consent forms -Conducted interviews/focus groups in Mandarin -Audio/video recorded with permission -Took observational field notes	Face-to-face in university meeting rooms; participants used think-aloud while describing human–computer collaborative classroom second language learning engagement experiences
2. Expert Review & Pilot	2 months	-Reviewed item pool independently -Rated each item’s relevance (CVI) -Provided written qualitative feedback (via email)	-Compiled expert ratings -Calculated I-CVI (>0.78 retained) and S-CVI (>0.80 retained)	Asynchronous digital (PDF review sheets sent via email with 2-week return deadline)
3. Pilot Testing	1 months	-Accessed questionnaire via Wenjuanxing QR code -Completed item pilot survey in computer lab (20 min) -6 participants engaged in think-aloud protocol (additional 15 min)	-Monitored lab session -Recorded think-aloud sessions -Analyzed pilot data for item difficulty, discrimination, and comprehension	Computer-mediated synchronous (controlled lab environment with researcher present)
4. Large-Scale Validation	3 months	-Scanned QR code distributed in class -Completed item survey on personal smartphones (15 min average) -Received automated attention-check items embedded within survey -Submitted survey + lottery entry	-Monitored response collection daily -Conducted data quality checks (attention checks, completion time < 7 min flagged) -Performed EFA/CFA -Administered criterion measures to subset	Self-paced, remote (participants could complete anywhere but during designated 1-week window; researcher available via WeChat for technical support)

**Table 3:** KMO and Bartlett’s Test of Sphericity Results.

Kaiser–Meyer–Olkin Measure of Sampling Adequacy		0.918
Bartlett’s Test of Sphericity	Approx. Chi-Square	4002.369
	df	171
	Significance	0.000

**Table 4:** Rotated Factor Loadings Matrix (N = 437).

Items	Factors				
	1	2	3	4	5
I5	0.810				
I3	0.746				
I1	0.733				
H4	0.671				
B2		0.702			
B1		0.699			
A2		0.657			
A1		0.618			
B3		0.610			
G3			0.745		
D2			0.721		
D1			0.714		
G4			0.648		
C2				0.824	
C1				0.795	
C3				0.736	
E2					0.749
E3					0.742
E1					0.692

**Table 5:** Results of Confidence Analysis (N = 273).

Factors	Items	Cronbach’s α	CR
Higher Order Thinking	A1	0.888	0.8831
	A2		
	B1		
	B2		
	B3		
Student–Teacher Interaction	C1	0.842	0.8425
	C2		
	C3		
Active Collaborative Learning	D1	0.873	0.8648
	D2		
	G3		
	G4		
Human–Computer Interaction	E1	0.849	0.8541
	E2		
	E3		
Enthusiasm for Chinese Language Learning	I1	0.867	0.8681
	I3		
	I5		
	H4		

**Table 6:** Results of Aggregation Validity Analysis.

Trails			Standard Estimate	AVE	CR
A1	<--	CS	0.741	0.602	0.883
A2	<--	CS	0.730		
B1	<--	CS	0.809		
B2	<--	CS	0.758		
B3	<--	CS	0.837		
C1	<--	ST	0.844	0.641	0.843
C2	<--	ST	0.773		
C3	<--	ST	0.783		
D1	<--	IC	0.730	0.616	0.865
D2	<--	IC	0.790		
G3	<--	IC	0.841		
G4	<--	IC	0.774		
E1	<--	CT	0.721	0.663	0.854
E2	<--	CT	0.833		
E3	<--	CT	0.880		
I1	<--	HI	0.795	0.6222	0.8681
I3	<--	HI	0.819		
I5	<--	HI	0.760		
H4	<--	HI	0.780		

Note. CS stands for higher-order thinking, ST stands for the student–teacher interaction, IC stands for active collaborative learning, CT stands for the human–computer interaction, and HI stands for enthusiasm for Chinese language learning.

**Table 7:** Criteria for Model Fitting Indexes.

Indexes	Adaptation Standards	
	Good	Acceptable
χ^2^/df	<3.0	<5.0
RMSEA	<0.08	<0.10
NFI	≥0.90	[0.70, 0.90)
PNFI	≥0.90	[0.70, 0.90)
CFI	≥0.90	[0.70, 0.90)
IFI	≥0.90	[0.70, 0.90)
TLI	≥0.90	[0.70,0.90)
PGFI	≥0.90	(0.60, 0.90)
GFI	≥0.90	(0.60, 0.90)
AGFI	≥0.90	(0.60, 0.90)

## Data Availability

The data that support the findings of this study are available from the corresponding author upon reasonable request.

## Appendix A

**Table A1:** Human–Computer Collaborative Classroom Second Language Learning Engagement Scale.

After each statement, there are seven numbers: (1) Strongly disagree, (2) Disagree, (3) Slightly disagree, (4) Slightly agree, (5) Moderately agree, (6) Agree, (7) Strongly agree.	
I5	I approve of the teacher’s teaching behaviors in the Chinese language classroom learning.
I3	Chinese language learning has improved my verbal and writing skills.
I1	The study of Chinese language learning has broadened my horizons and enriched my cultural knowledge.
H4	I am interested in what I am learning in the Chinese language classroom.
B2	I can guess the meaning of words of the Chinese language or infer the general meaning of a passage with the help of context or background.
B1	To memorize the vocabulary of the Chinese language learning, I try to use the vocabulary in my writing or oral expressions.
A2	I know exactly what learning goals I want to achieve in Chinese language classroom.
A1	I have an explicit requirement to improve my reading and writing skills in Chinese language learning.
B3	I will relate the prior experience to what I have learned when learning the Chinese language.
G3	I will take the initiative to participate in group activities and discussions in the Chinese language classroom.
D2	In the Chinese language classroom, I often collaborate with my classmates.
D1	Many students were willing to be in my group when grouped in the Chinese language classroom.
G4	I am active and positive in the Chinese language classroom.
C2	I prefer to interact and discuss with the teacher instead of interacting with my classmates.
C1	Outside of class time, I will discuss problems encountered in the Chinese classroom with teacher.
C3	When communicates online, the teacher is always prompt in answering our questions about Chinese language learning.
E2	In ICT environment, I will use search engines to find relevant information from the internet on the topics discussed in class.
E3	Information technology used in ICT environment will deepen my understanding and knowledge of learning the Chinese language.
E1	In the ICT environment, I will use the Chinese language to communicate and discuss ideas in online forums and chat tools.
